# Validity of self-reported number of teeth in middle-aged Finnish adults: the Northern Finland Birth Cohort Study 1966

**DOI:** 10.1186/s12903-018-0666-4

**Published:** 2018-12-11

**Authors:** Toni Similä, Pentti Nieminen, Jorma I. Virtanen

**Affiliations:** 1 Research Unit of Oral Health Sciences, Faculty of Medicine, University of Oulu, P.O. Box 5000, 90014 Oulu, Finland; 2 Medical Research Center Oulu, Oulu University Hospital, P.O. Box 5000, 90029 Oulu, Finland; 3 Medical Informatics and Data Analysis Research Group, University of Oulu, P.O. Box 5000, 90014 Oulu, Finland; 4 Institute of Dentistry, University of Turku, 20014 Turku, Finland

**Keywords:** Adult, Self report, Tooth, Tooth loss, Validity

## Abstract

**Background:**

We examined the validity of self-reported number of teeth in middle-aged adults by using representative cohort data to compare corresponding self-reported and clinical values.

**Methods:**

This validity study is part of the representative 46-year-old follow-up of the Northern Finland Birth Cohort 1966 (NFBC1966) Study. Mailed questionnaires (*n* = 5950) requested information on self-reported number of teeth and background variables (education, tooth brushing and smoking), while clinical oral health examinations (*n* = 1891) assessed the number of teeth (the ‘gold standard’). The main analyses compared the self-reported and clinical values for the number of teeth in 1669 participants. Scatterplot and Bland-Altman plot served for visual analyses, and alternative correlation coefficients (Pearson, Spearman, intraclass) for numerical comparisons separately for men and women, with stratification according to background variables.

**Results:**

The clinical assessment revealed that the mean value for the number of teeth was 27.46 (SD = 2.38), while the corresponding value based on self-reported information was 27.48 (SD = 2.78). According to the Bland-Altman plot, the mean difference between the clinical and self-reported values was − 0.02 (95% limits of agreement, LoA: − 3.37 to 3.32). The observed ranges of intraclass correlation coefficients (ICC) among men and women were 0.72 to 0.95 and 0.72 to 0.85, respectively, depending on the background variables.

**Conclusions:**

Self-reported number of teeth in middle-aged Finnish adults agreed closely with the corresponding clinical measure.

## Background

Tooth loss, assessed by the number of remaining/missing teeth (whether self-reported or clinically assessed), is a simple and widely used measure of oral health status in dental research [1]. Especially among middle-aged and older people, who tend to have fewer remaining teeth with increased age, tooth loss is an optimal outcome measure of oral health status when its association with general diseases (e.g. diabetes, cardiovascular disease) or health behavior (e.g. smoking, alcohol use) is a subject of interest [2–4]. Professional oral health examinations to assess the oral health status of the subject are expensive, time consuming and labor intensive. In contrast, number of teeth is a simple measure which seldom requires professional clinical assessment and is easily incorporated into health questionnaires or interviews [3].

In general, self-reported number of teeth is an acceptable and easily obtainable substitute for corresponding objective clinical measures. However, some previous studies have reported a tendency towards underestimation of the number of teeth in self-reports compared to trained clinical assessments [5, 6]. Moreover, number of teeth validity seems to vary according to certain characteristics (e.g. age, oral health behavior, number of remaining teeth) [5–7]. Especially aging predicts prosthetic restorations, which make accurate self-reporting more difficult.

Middle-aged Finns enjoy good oral health, thanks mainly to the availability of subsidized dental care from childhood [8]. Correspondingly, our earlier study found that 60% of the sample of 46-year-old Finns reported having at least 28 teeth in their mouth [3]. The study reported that the validity of dichotomous self-reports (0–27 or 28–32 remaining teeth) was substantial (Cohen’s kappa 0.78) compared to professional clinical assessment, with 82% of the participants’ self-reported values for the number of teeth falling within a one-tooth margin of error from the clinically assessed value. However, Similä et al. [3] only explored the validity of self-reported number of teeth to support the main analyses of their study.

Previous studies have offered only limited evidence of the validity of self-reported measures in confined study populations, hence the need for comprehensive studies among different populations. Moreover, studies have neglected to investigate the validity of self-reports among different sub-groups based on background characteristics such as socioeconomic status and health behavior. Therefore, in this study, we examine the validity of self-reported (remaining) number of teeth in middle-aged Finns with good oral health. We hypothesize that self-reported values will agree closely with the corresponding clinical assessment in this study population. In addition, this study assesses the possible effect of background characteristics on the validity of self-reported information.

## Methods

### Study subjects

This cross-sectional validity study used data from the longitudinal Northern Finland Birth Cohort Study 1966 (NFBC1966), which consists of a comprehensive sample of individuals from the two northernmost provinces of Finland (Lapland and Oulu) whose expected year of birth was 1966 (12,068 mothers, 12,231 children, 96.3% of all births in this region) [9]. Regular monitoring of the cohort members since their mothers were pregnant has provided information across several life stages. The Ethics Committee of the Northern Ostrobothnia Hospital District in Oulu, Finland approved the study protocol, which adhered to the principles of the Declaration of Helsinki. The participants’ participation was voluntary, and all provided their written informed consent. The data were handled on a group level only; identification codes replaced their personal information.

This study includes data from the 46-year follow-up (carried out in 2012–2014) and consisted of a mailed survey (between April 2012 and October 2013) and a comprehensive in-person clinical health examination. The questionnaires and invitations to health examinations were mailed to all who lived in Finland and whose addresses were known at the beginning of 2012 (*n* = 10,321). Subjects living in municipalities within 100 km of the city of Oulu (Oulu subpopulation, *n* = 3135) were invited to more thorough health examinations, which included a full inspection of the mouth and teeth (from April 2012 to June 2013).

Firstly, mailed questionnaires (*n* = 5950, participation rate 58%) offered information on self-reported number of teeth (not excluding wisdom teeth) and background variables (education, tooth brushing and smoking). Oral health examinations then included a clinical assessment of the number of teeth (the ‘gold standard’) for the subsample (*n* = 1891, participation rate 60%). Our analyses focused on the comparison between self-reported and clinical values for the number of teeth (*n* = 1669). On average, five months passed between the two assessments.

### Number of teeth

The mailed survey inquired about the participant’s number of teeth at the age of 46 with the question “What is the total number of teeth in your mouth?”, so it was impossible to distinguish between wisdom teeth and other teeth. No additional instructions were given. The clinical oral examination covered professional assessments for each tooth separately [10]. A total of seven trained and calibrated dentists carried out the examinations and counted the number of missing teeth (including wisdom teeth) in each participant. During the examinations, a trained dental nurse assisted and registered the findings into electronic form. With regard to the validity assessment of this clinical measure, Cohen’s kappa values for inter- and intra-examiner agreements on the tooth level were 1.00 and 0.97, respectively [11, 12]. For the analyses, we created a variable for the number of teeth in each participant (32 – “number of missing teeth”).

### Background variables

Gender, education, tooth brushing frequency and smoking status served as background variables. Except for gender, the mailed questionnaire requested information on these variables. We formed a three-class ordinal variable from the participant’s reported level of education at the age of 46 as follows: ‘basic education’ included those who had not graduated from high school and had no formal vocational qualification, ‘secondary education’ included those who had graduated from high school or vocational school, and ‘higher education’ included participants with a university degree or who had graduated from a polytechnic or equivalent school.

We dichotomized the response information for the question on the frequency of tooth brushing based on the general recommendation to brush twice daily: ‘once daily or less’ or ‘at least twice daily’ [13].

Several questions asked about previous and current smoking habits at the age of 46 [3]. We aggregated the answers to these questions to determine the participants’ smoking status as current, former or never smokers. Here, ‘current smokers’ were those who reported smoking at least occasionally; ‘former smokers’ included those who had smoked daily for at least one year, but had quit smoking and were not smokers at the time of the study; ‘never smokers’ included all participants who had smoked daily for less than one year in their lifetime and were not smokers at the time of the follow-up.

### Statistical analysis

**Fig. 1:**
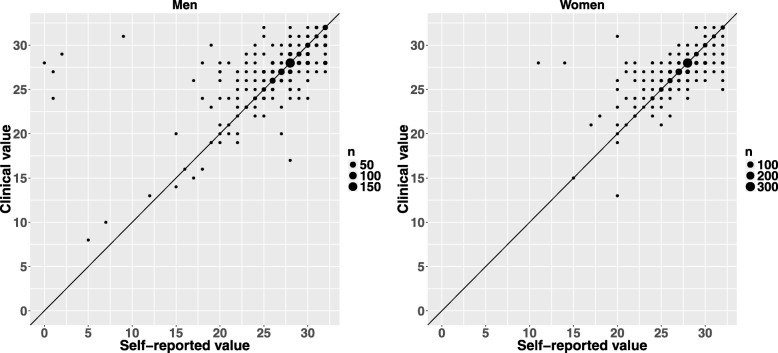
The agreement between the self-reported and clinically assessed number of teeth among men (*n* = 745) and women (*n* = 929)

We conducted all validity analyses (except for Fig. 1) by excluding participants (considered outliers) whose self-reported values of number of teeth differed drastically (≥ 20 teeth; *n* = 5) from the corresponding objective clinical value.

Scatterplot served for basic visual comparison between self-reported and clinical values for the number of teeth. Then, for a more sophisticated approach, Bland-Altman plot was utilized [14].

For the numerical comparison between self-reported and clinical values for the number of teeth, we carried out the analyses separately for men and women based on our earlier findings [2, 3, 11]. We calculated alternative correlation coefficients (Pearson, Spearman, intraclass) among men and women with stratification according to background variables: education, tooth brushing frequency and smoking status. Intraclass correlation coefficient (ICC) estimates were based on single-rating (type), absolute-agreement (definition), two-way mixed effects model [15–17].

We used the statistical package R environment version 3.3.2 for all statistical analyses [18]. The analyses made use of following packages: BlandAltmanLeh, ggExtra, ggplot2, gridExtra, irr.

## Results

According to the objective clinical assessment, the mean value for the number of teeth was 27.46 (standard deviation, SD = 2.38), while the corresponding value based on self-reported information was 27.48 (SD = 2.78) and the value for the difference, between these two, was − 0.02 (SD = 1.71). These values for men were 27.48 (SD = 2.78), 27.48 (SD = 3.19) and − 0.004 (SD = 1.89), respectively; for women, 27.44 (SD = 1.99), 27.48 (SD = 2.41) and − 0.04 (SD = 1.55), respectively.

**Fig. 2:**
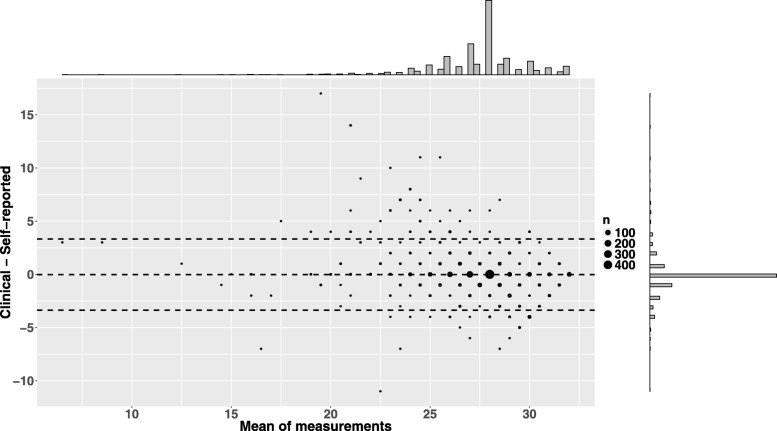
The Bland-Altman plot for the agreement between the self-reported and clinically assessed number of teeth (*n* = 1669). The plot includes horizontal lines (three in total) for the mean difference of the measures and 95% limits of agreement (LoA), which are calculated as ±1.96*SD away from the mean difference of the measures

The scatterplots indicate the degree of agreement between the self-reported and clinically assessed number of teeth among men and women (Fig. 1). Five outliers are clearly visible among the men. Otherwise, the pattern between self-reported and clinical values among men and women is consistent. Figure 2 visualizes the distributions and agreement of self-reported and clinical measures with histogram-style marginal graphs in the Bland-Altman plot. The mean difference between the values was − 0.02 (LoA: − 3.37 to 3.32).

Table 1 shows basic distributions and the comparison between different correlation coefficients according to background variables among men. The observed ranges of the Pearson, Spearman and intraclass correlation coefficients, depending on the background variables, were 0.74 to 0.96, 0.74 to 0.91 and 0.72 to 0.95, respectively. The values for the correlation coefficients seemed to vary according to education level and smoking status. For education, the observed agreement was the highest among those with basic education level (*r* = 0.96, *ρ* = 0.91, ICC = 0.95). Table 2 provides corresponding information about women. The observed ranges of the Pearson, Spearman and intraclass correlation coefficients, depending on the background variables, were 0.73 to 0.86, 0.81 to 0.88 and 0.72 to 0.85, respectively. The values for the correlation coefficients varied less among the women than among the men. For smoking status, the observed agreement was the highest among former smokers (*r* = 0.86, *ρ* = 0.85, ICC = 0.85).

**Table 1:** Alternative correlation coefficients (Pearson r, Spearman ρ, intraclass ICC) for the self-reported and clinically assessed number of teeth among men

	*n*	%	r	ρ	ICC
Education (*n* = 730)					
Basic	45	6	0.96	0.91	0.95
Secondary	322	44	0.80	0.74	0.79
Higher	363	50	0.74	0.79	0.72
Tooth brushing frequency (*n* = 738)					
Once daily or less	359	49	0.81	0.78	0.80
At least twice daily	379	51	0.80	0.79	0.80
Smoking status (*n* = 680)					
Never	299	44	0.75	0.79	0.74
Former	208	31	0.79	0.80	0.79
Current	173	25	0.87	0.77	0.86
Total	740	100	0.81	0.78	0.80

**Table 2:** Alternative correlation coefficients (Pearson r, Spearman ρ, intraclass ICC) for the self-reported and clinically assessed number of teeth among women

	*n*	%	r	ρ	ICC
Education (*n* = 916)					
Basic	34	4	0.83	0.88	0.80
Secondary	260	28	0.76	0.82	0.75
Higher	622	68	0.76	0.82	0.75
Tooth brushing frequency (*n* = 929)					
Once daily or less	187	20	0.80	0.81	0.77
At least twice daily	742	80	0.75	0.83	0.74
Smoking status (*n* = 859)					
Never	481	56	0.75	0.83	0.74
Former	191	22	0.86	0.85	0.85
Current	187	22	0.73	0.82	0.72
Total	929	100	0.77	0.82	0.76

## Discussion

This study shows that self-reported tooth loss is a reliable measure in middle-aged Finns. Overall, agreement between self-reported and clinical measures was high among men and women with slight variation by gender, which agrees with our hypothesis. However, the values for the correlation coefficients varied by background characteristics such as education level and smoking status.

Few studies have focused specifically on the validity of self-reported number of teeth. Of two Japanese studies on this topic, the earlier one was based on a sample of 40- to 56-year-old dentate community residents (*n* = 1152) who completed self-administered questionnaires and underwent dental examinations at local dental offices [6]. The authors reported that the values for the Pearson and intraclass correlation coefficient were 0.80 and 0.78, respectively, for the entire sample. Our results were similar, as we observed overall values for the Pearson, Spearman and intraclass correlation coefficients to be 0.79, 0.80 and 0.78, respectively. The more recent Japanese study examined participants 35 years of age and older (*n* = 1501) who took part in a self-administered questionnaire and clinical examination of their dental status [5]. The study found that the Spearman correlation coefficients for men and women were 0.70 and 0.67, respectively. However, we obtained slightly higher values for the Spearman correlation coefficients among men and women (0.78 and 0.82) than those in the study.

Most studies that use self-reported data have reported slight underestimations of the self-reported number of teeth [5, 6, 19]. In contrast, a study of the adult Swedish population reported overestimation of the number of teeth, especially among older participants [7]. Unlike these studies, we observed no tendency towards either under- or overestimation of the self-reported number of teeth when compared to corresponding objective clinical measure. However, the self-reported number of teeth among men showed notable underestimation until we removed the outliers from the analyses.

Scatterplots in Fig. 1 provided the opportunity to identify the outliers, values that fall far outside the mainstream values [20]. We excluded five cases which we suspected to be clearly incorrect recordings or the result of misunderstanding of the question related to the number of teeth (likely reported as the number of missing teeth instead). Thus, we avoided information bias, which might otherwise have occurred due to incorrect measurements of the number of teeth [21]. In any case, the low number of excluded cases in this study supports the usefulness of the self-reported measure in comparable study populations.

Previous studies have generally neglected to investigate the validity of self-reported number of teeth according to basic background factors (i.e. socioeconomic status and smoking) known to associate with oral health. Matsui et al. [5] reported correlation values between self-reported and clinically assessed number of teeth according to tooth brushing frequency (‘Once a day’, ‘Twice a day’, ‘3 or more times a day’). They merely noted that the correlations were statistically significant for each tooth brushing category, and that the correlation values showed little variation between categories. Similarly, we observed no variations in correlations between the categories of our dichotomized tooth brushing frequency variable. In addition to tooth brushing, we investigated the correlations according to education level and smoking status.

Our findings imply that agreement between self-reported and clinical measures could be higher among those with a basic education level than among those with a higher education level. In addition, different correlation coefficients showed mutually inconsistent values across the categories of smoking status among men. We argue that agreement between self-reported and clinical values is in some way related to the degree of tooth loss. Previous studies that examined data from the 46-year-old NFBC1966 study population have demonstrated a strong association between tooth loss and current smoking or low education level [3, 11]. Consequently, those who have fewer teeth and possibly other complications as well, ought to be better aware of their oral health status and, might report their number of teeth accurately.

To our knowledge, this was the first study to assess the validity of self-reported number of teeth in a Finnish study population. The NFBC1966 46-year follow-up provided a representative sample of the middle-aged Finnish population, with its good level of oral health.

We used three alternative correlation coefficients (Pearson, Spearman, intraclass) to examine the validity of self-reported data. Therefore, we were able to strengthen our findings and provide a more thorough comparison with earlier studies that used differing measures of correlation [5, 6]. In addition, our use of a Bland-Altman plot offers a novel approach (from the common scatterplot) to visually assessing the validity of self-reported number of teeth.

Some of the previous studies explored the validity of self-reported number of teeth among different age groups [5, 7]. We were able to study the validity only among 46-year-olds, which limits the generalizability of our results to other age groups. On the other hand, the Japanese studies took into account the possibility that the validity of self-reporting could ultimately depend on the actual number of teeth [5, 6]. Although considering this connection possible, we did not assess the validity of self-reporting based on the actual number of teeth due to the overall high number of teeth values in this study population, with its good level of oral health, as doing so would have made it difficult to define comparable groups.

One of the most important factors influencing the validity of self-reporting is the assessment method itself (e.g. mailed questionnaire, telephone survey, face-to-face interview). In the NFBC1966 46-year-old follow-up, information on the self-reported number of teeth was collected through a mailed survey containing the question: “What is the total number of teeth in your mouth?” This question is very simple and offers no additional guidance on whether to include, for instance, implants and roots in the calculation. It also assumes that respondents will by default include wisdom teeth in the calculation. Despite these shortcomings, however, the participants reported their number of teeth quite accurately.

## Conclusions

In middle-aged Finnish adults, self-reported number of teeth agreed closely with the corresponding clinical measure. Although some validity problems remain among certain sub-groups, self-reported information can replace clinical assessment as an easily obtainable cost-effective alternative for studies that use tooth loss measures in Nordic study populations. Using self-reported tooth loss measures as a primary screening tool in a risk factor questionnaire enables the identification of subjects who might benefit from further examinations.

## Abbreviations

ICC: Intraclass correlation coefficient
LoA: Limits of agreement
NFBC1966: The Northern Finland Birth Cohort 1966
SD: Standard deviation
